# Climate change indicators dataset for coastal locations of the European Atlantic area

**DOI:** 10.1016/j.dib.2022.108339

**Published:** 2022-06-01

**Authors:** Bassel Habeeb, Emilio Bastidas-Arteaga

**Affiliations:** a Institute for Research in Civil and Mechanical Engineering (GeM UMR CNRS 6183), University of Nantes, France; b Laboratory of Engineering Sciences for Environment (LaSIE UMR CNRS 7356), La Rochelle University, France

**Keywords:** Climate indicators database, Atmospheric indicators, Oceanic indicators, Climate change, Atlantic area, Coastal infrastructure assets

## Abstract

Over time, considerable changes in the earth's climate have always occurred due to a wide variety of natural processes. During the last century, these natural changes have all been accelerated by global warming, which has been driven by human activities. Climate change leads to wide variations in environmental variables such as temperature, relative humidity, carbon dioxide, etc. These changes could adversely affect the performance, serviceability, and safety of infrastructure assets. The challenge, therefore, is to not only understand the effect of extreme events and their links to climate change, but also to obtain data that could be used for assessing long-term gradual effects affecting infrastructure assets. In this paper is presented a climate indicators database that was collected and provided in an excel format. This database could be used for assessing the durability, vulnerability, and cost-effectiveness of adaptation measures for coastal infrastructure assets. The database contains information for specific coastal locations placed in five European countries: Caxias (Portugal), Saint Nazaire (France), Vigo (Spain), Brighton (UK), Dublin and Cork (Ireland). The database includes atmospheric, and oceanic indicators, as well as and the flow of rivers. It covers a time series of up to 2100 with various representative concentration pathways and climate models.

## Specifications Table

**Table:** 

Subject	Civil and Structural Engineering
Specific subject area	Atmospheric and oceanic climate variables and river flow for infrastructure vulnerability assessment for several locations in the European Atlantic area
Type of data	Tables
How data were acquired	Data was extracted from: World Climate Research Program, Coupled Model Inter-comparison Project 5, Copernicus Climate Change Service, Coordinated Regional Climate Downscaling Experiment
Data format	Filtered
Description of data collection	The main conditions for collecting data were: (1) to select coastal locations in the European Atlantic Area with different weather conditions; (2) to choose models and resolutions that are appropriate for lifetime and vulnerability infrastructure assessment; (3) to select climate parameters affecting the durability and safety of infrastructures in coastal areas; (4) to provide information in an excel format that could be easily used by researchers and companies; (5) to deliver historical data and predictions for several climate change scenarios extracted from several climate models.
Data source location	First location of interest: City/Town/Region: Saint Nazaire/Loire-Atlantique/Pays de la Loire. Country: France. Latitude and longitude: 47.31° N, 2.17° W
	Second location of interest:City/Town/Region: Vigo/Galicia/Pontevedra. Country: Spain. Latitude and longitude: 42.19° N, 8.78° W
	Third location of interest: City/Town/Region: Caxias/Oeiras /Lisbon. Country: Portugal. Latitude and longitude: 38.65° N, 9.3°W
	Fourth location of interest: City/Town/Region: Brighton/East Sussex/South East England. Country: United Kingdom. Latitude and longitude : 50.85° N, 0.11° W
	Fifth location of interest: City/Town/Region: Dublin/Leinster/Eastern Ireland. Country: Ireland. Latitude and longitude: 53.29° N, 6.3° W
	Sixth location of interest: City/Town/Region: Cork/Munster/South West Ireland. Country: Ireland. Latitude and longitude: 51.87° N, 8.54° W
	Primary data sources and models: Regional atmospheric climate models: (RCA4, HIRAM5, WRF381P, CCLM4–8–17, RACMO22E, REMO2015) Global oceanic climate models: (CANESM2, MOHC—HadGEM2, NIMR-KMAHadGEM2-AO, ERA5) River flow regional model: (IMPACT2C)
Data accessibility	Direct link to the dataset: https://sirma-project.eu/dissemination/climate-change-indicators-database/

## Value of the Data

• The database consists of atmospheric and oceanic datasets that provide an objective basis for understanding and predicting future evolutions, and related effects for the built environment, of atmospheric, and oceanic variables or river flow in specific locations of the European Atlantic area.
• This database was mainly proposed for research institutions and enterprises interested in estimating the durability, consequences, vulnerability, and cost-effectiveness of adaptation measures for infrastructure assets and buildings in specific locations of European Atlantic regions [1,2]. However, other sectors could also take advantage of this database.
• The database is provided in an excel format that could be used easily. Information from specific locations, variables, climate change scenarios, and models is available for assessing climate change effects on infrastructure assets and formulating adaptation strategies. Comprehensive lifetime assessment models considering time-variant climatic inputs could be used towards these aims. Moreover, it is valuable to develop stochastic predicting models.

## 1 Data Description

The database includes atmospheric, oceanic and river flow datasets. The climate indicators availability in the database is described in Table 1, Table 2, Table 3. The climate change indicators database comprises a variety of climate models, in which each model has specific driving methods to generate the data e.g., ensemble, data source, Institution, Jet stream, influence, aerosols, forcing, initial state of run, etc. The availability of different models provides one idea of the levels of uncertainty of the model that is useful for consequence analysis or lifecycle assessment. The climate change indicators database aims to cover the historical period and several climate change scenarios, as follows: RCP 2.6, RCP 4.5, RCP 6.0, and RCP 8.5 [3,4].

**Table 1:** Oceanic dataset availability.

				Sea Surface Temperature	Sea Surface Salinity	Sea Water X Velocity	Sea Water Y Velocity	Sea Water Pressure at Sea Water Surface	Significant height of combined wind waves and swell	Mean wave period	Sea Surface Height Above Geoid
				
Model	Start period	End period	Projections	Celsius Degree	PSU	*m/s*	*m/s*	Decibar	m	*sec*	m
CANESM2	Jan. 1850	Dec. 2100	RCP 2.6 RCP 4.5 RCP 8.5	+	+	+	+				+
MOHC—HadGEM2	Dec. 1859	Dec. 2099	RCP 2.6 RCP 4.5 RCP 6.0 RCP 8.5	+	+						+
NIMR-KMA HadGEM2-AO	Jan. 1860	Dec. 2100	RCP 2.6 RCP 4.5 RCP 6.0 RCP 8.5	+	+	+	+	+			
ERA5	Jan. 1979	Dec. 2099	No projections						+	+

**Table 2:** Atmospheric dataset availability.

				Near Surface Air Temperature	Near Surface Relative Humidity	Precipitation	Daily Mean Near-Surface Wind Speed
				
Model	Start period	End period	Projections	Celsius Degree	%	kg m^-2^ s^-1^	*m/s*
RCA4	1/01/1971	31/12/2100	RCP 2.6 RCP 4.5 RCP 8.5	+	+	+	+
HIRHAM5	26/12/1950	27/06/2098	RCP 4.5 RCP 8.5	+	+	+	+
WRF381P	01/01/1951	30/11/2099	RCP 8.5	+			
CCLM4–8–17	01/01/1951	31/12/2100	RCP 4.5 RCP 8.5	+			
RACMO22E	26/12/1950	27/06/2098	RCP 2.6 RCP 4.5 RCP 8.5	+	+	+	+
REMO2015	12/01/1951	11/01/2101	RCP 8.5	+	+	+	+

**Table 3:** River dataset availability.

Model	Start period	End period	Projections	River Flow
				*m^3^/s*
IMPACT2C	1/01/1979	31/12/2100	RCP 2.6 RCP 4.5 RCP 8.5	+

The resolution of the climate models varies as shown in Table 4, resulting in differences in the regions covered by each model. The atmospheric dataset is obtained from high-resolution regional models and provides representative information for the locations of interest. However, the oceanic dataset should be validated as it is obtained from low-resolution global climate models. The resolution of the river flow could cover several rivers in the region. Therefore, some post-treatment processing of the data is required to determine the river flow for a specific river.

**Table 4:** Models' resolution.

		Approx. resolution (km)	
Dataset	Model	Latitude	Longitude
Ocean	CANESM2	103.2	156.1
	MOHC—HadGEM2	111.0	111.0
	NIMR-KMAHadGEM2-AO	111.0	111.0
	ERA5	55.5	55.5

Atmosphere	RCA4	12.2	12.2
	HIRHAM5	12.2	12.2
	WRF381P	12.2	12.2
	CCLM4–8–17	12.2	12.2
	RACMO22E	12.2	12.2
	REMO2015	12.2	12.2

River	IMPACT2C	55.5	55.5

The selected geographical locations are located alongside the European Atlantic Ocean area (Table 5). The extraction of the database is based on the nearest point to the model's coordinates. The region covered in each model is provided by the resolution distribution worksheets available in the document D.SIRMA-WP4-3.2-RD. A sample of this file is presented in Table 5.

**Table 5:** Resolution distribution for the atmospheric dataset.

	Resolution Distribution (Degree)				Extraction point (Degree)	
City	Latitude distribution-1	Latitude distribution-2	Longitude distribution-1	Longitude distribution-2	Latitude	longitude
Caxias	38.595	38.705	9.245	9.355	38.65	9.3
Saint Nazaire	47.255	47.365	2.115	2.225	47.31	2.17
Vigo	42.135	42.245	8.725	8.835	42.19	8.78
Brighton	50.795	50.905	0.055	0.165	50.85	0.11
Dublin	53.235	53.345	6.245	6.355	53.29	6.3
Cork	51.815	51.925	8.485	8.595	51.87	8.54

The Model description Excel Worksheets D.SIRMA-WP4-3.1-MD describes the Dataset Metadata for each climate model, provides the data source, and information for each climate model. Table 6 represents a sample of these Excel Worksheets, in which the indicated forcing factors are defined in the Excel Worksheet D.SIRMA-WP4-2.1-VFD.

**Table 6:** Dataset metadata sample.

	Dataset Metadata
Climate model	HadGEM2-AO
Project	CMIP5
Product	Output1
Institute	NIMR-KMA
Experiment	Historical & All RCP
Time-frequency	Monthly
Reaim	Ocean
Ensemble	r1i1p1
Version	20,121,018
Data source	HadGEM2-AO r6.6.3 (2010): atmosphere: HadGAM (HadGAM2, N96L38); ocean: HadGOM (HadGOM2, 1 × 1L40, increased resolution at Equator); sea ice: part of HadGOM2; land: MOSES-2
Forcing	Nat, Ant, GHG, SA, Oz, LU, Sl, Vl, SS, Ds, BC, MD, OC

## 2 Experimental Design, Materials and Methods

The main conditions for collecting data are summarized as follows. We focused on coastal infrastructure located in the European Atlantic Area which is the region of study of the SIRMA project. The database in intended to be used for evaluating the effects and proposing adaptation solutions for infrastructure assets and buildings. Therefore, the conditions to select and extract parameters were defined based on the most common durability and vulnerably issues affecting these assets, e.g., corrosion, chloride ingress into concrete, extreme winds, erosion, sea-level rise, etc.

The choice of a climate model with a very large resolution could lead to wrong lifetime or vulnerability infrastructure assessments. Therefore, we considered models and resolutions that are representative of different infrastructure assets. Historical data and predictions for several climate change scenarios are also considered lifetime or vulnerability assessments for existing and new infrastructure under past and future weather conditions. The inclusion of several climate models in the database allows to consider the model uncertainty for the infrastructure assessments.

The information was extracted from three main databases: World Climate Research Program, Coupled Model Inter-comparison Project 5, Copernicus Climate Change Service, Coordinated Regional Climate Downscaling Experiment. However, the information given in these databases is not easy to use for researchers and companies. Therefore, this database provides a friendly website application that allows to download data in Excel format depending on the requirements of the user.

This database was extracted and prepared by writing and using a numerical script using R software. Those scripts are capable of:

1- Extracting variables for a region determined by the model's resolution.
2- Describing each model dataset Metadata to distinguish the measurement variation of a common variable (e.g., experiment, aim, frequency, start date, institution, project, product, source, forcing, ensemble, Version, driving model).

## Ethics Statement

This database did not involve the use of human subjects, animal experiments, nor data collected from social media platforms.

## CRediT authorship contribution statement

**Bassel Habeeb:** Conceptualization, Methodology, Software, Data curation, Writing – original draft. **Emilio Bastidas-Arteaga:** Conceptualization, Methodology, Supervision, Validation, Funding acquisition, Writing – review & editing.

## Declaration of Competing Interest

The authors declare that they have no known competing financial interests or personal relationships that could have appeared to influence the work reported in this paper.

## Data Availability

Climate change indicators database (Reference data) (SIRMA project website).
